# Long-term outcomes after endoscopic sleeve gastroplasty: impact of clinician follow-up and lifestyle engagement

**DOI:** 10.1007/s00464-026-13151-3

**Published:** 2026-07-22

**Authors:** Wissam Ghusn, Ayah Matar, Karim Al Annan, Lea Sayegh, Khushboo Gala, Omar M. Ghanem, Daniel B. Maselli, Eric J. Vargas, Barham K. Abu Dayyeh, Andrew C. Storm

**Affiliations:** 1 Department of Internal Medicine, Boston University, Boston, MA USA; 2 Division of Gastroenterology and Hepatology, Mayo Clinic, Rochester, MN USA; 3 Department of Surgery, Mayo Clinic, Rochester, MN USA; 4 Division of Gastroenterology and Hepatology, Cedars-Sinai Medical Center, Los Angeles, CA USA; 5 Section of Gastroenterology, Wake Forest School of Medicine, Winston Salem, NC USA

**Keywords:** ESG, Weight loss, Exercise, Obesity management

## Abstract

**Background:**

Bariatric interventions rely on lifestyle changes that benefit from long-term follow-up and patient engagement. Mid- and long-term outcomes following endoscopic sleeve gastroplasty (ESG) are heterogeneous and likely multifactorial. While the literature has explored patient demographic and anthropometric influence, as well as procedural technique, on post-ESG weight loss, there are knowledge gaps about the influence of aftercare programs. We hypothesize that clinician follow-up intensity and exercise participation correlate with weight loss after ESG.

**Methods:**

We conducted a retrospective cohort study of adults who underwent ESG at a tertiary academic center with expertise in bariatric endoscopy. Patients with at least two years of follow-up and complete weight and behavioral data were included. Follow-up intensity was defined by the number of post-ESG physician or advanced practice clinician visits and categorized as close (≥ 5 visits) or limited (< 5 visits). The primary outcome was total body weight loss (TBWL%) at the most recent follow-up visit; the secondary outcome was the association between exercise participation and TBWL%.

**Results:**

A total of 56 individuals met inclusion criteria (mean age 49.6 years, 82.2% female). The mean duration of follow-up was 29.0 months. Baseline demographic and clinical characteristics were similar between groups. Patients with close clinician follow-up achieved significantly greater TBWL compared with those with limited follow-up (− 13.7 ± 11.2% vs. − 6.7 ± 8.2%, *p* = 0.01). In categorical analyses, a higher proportion of the close follow-up group achieved ≥ 10, ≥ 15, and ≥ 20% TBWL. Exercise participation was reported by 69.7% of participants and was associated with numerically higher but nonsignificant TBWL (*p* = 0.55).

**Conclusions:**

Heightened clinician follow-up after ESG was associated with approximately twice the magnitude of long-term weight loss. These findings emphasize that structured, ongoing engagement from clinical team members, as opposed to solely nutritional team members, is a critical determinant of durable success after ESG.

**Supplementary Information:**

The online version contains supplementary material available at https://doi.org/10.1007/s00464-026-13151-3.

Obesity is a chronic, relapsing disease that affects more than 40% of adults in the United States and contributes substantially to cardiometabolic morbidity, reduced quality of life, and healthcare costs [1, 2]. Traditional lifestyle interventions (i.e., dietary modification, behavioral therapy, and exercise) remain foundational but often yield modest and transient weight loss in the absence of ongoing support and close follow-up [3]. Incretin-mimetics have revolutionized obesity care [4, 5] but face challenges of high discontinuation rates [6], dose-limiting gastrointestinal intolerance [7], and barriers to access [8]. Metabolic and bariatric surgery currently provides the most significant weight loss and metabolic improvement [9] but is underutilized due to perceived risk and limited accessibility [10].

Endoscopic bariatric therapies (EBTs) have emerged as a critical, minimally invasive alternative and complement to pharmacologic and surgical options for obesity [11]. Among these, endoscopic sleeve gastroplasty (ESG) has become the most widely adopted, owing to its favorable safety profile, outpatient feasibility, and reproducible efficacy [12]. ESG facilitates approximately 15–20% total body weight loss (TBWL) at one year and demonstrates sustained metabolic benefits up to five years [9, 13]. The procedure’s favorable safety profile and recovery time have left to increased patient acceptance in those disinclined to pursue surgical weight loss [14, 15].

Weight loss after bariatric medications and interventions is heterogeneous, and treatment response characteristically presents as a bell curve, with considerable effort spent optimizing therapies to shift this curve rightward toward greater and sustained weight loss [16]. In keeping with this phenomenon, weight trajectories after ESG vary markedly, with some patients maintaining significant TBWL for years and others experiencing early plateau or weight recurrence [17]. Patient baseline characteristics, procedure technical factors, and changes to gastric motor physiology have been explored as factors that enhance weight loss after ESG [18–21]. Evidence from pharmacologic and surgical cohorts underscores that structured, frequent follow-up enhances adherence to dietary and lifestyle recommendations and predicts greater long-term weight maintenance [22]; however, systematic data on how follow-up intensity influences ESG outcomes remain limited to small cohorts and requires further exploration [23]. Furthermore, these follow-up studies typically group follow-up with any team member (e.g., physician, advanced practice provider, dietician) together. Given the multidisciplinary nature of obesity management and the proven need for frequent touchpoints for patient support, it remains unclear if clinical team member availability after ESG, beyond the essential initial weeks of recovery symptom and safety assessment, is influential for weight loss success. Understanding this would help better structure aftercare programs and post-procedure counseling.

Physical activity is an another cornerstone of comprehensive obesity management. Regular exercise promotes preservation of lean body mass, improves cardiorespiratory fitness, and augments metabolic health independent of weight loss [24]. Despite these benefits, exercise adherence after ESG and its impact on sustained weight reduction are poorly characterized in real-world settings.

Given these knowledge gaps, we sought to examine the relationship between post-procedure follow-up intensity, exercise engagement, and long-term weight outcomes in patients undergoing ESG.

We hypothesized that patients receiving close, structured clinical follow-up and participating in regular exercise programs would achieve superior and more durable TBWL, emphasizing that the success of ESG extends beyond procedural innovation to the quality and consistency of post-intervention care.

## Methods

### Study design and population

We conducted a retrospective cohort study of adults who underwent ESG for weight management at a tertiary academic medical center. All ESG procedures were performed by experienced bariatric endoscopists using the OverStitch endoscopic suturing system (Apollo Endosurgery, Austin, TX, USA) following a standardized institutional technique. Procedures were performed using a full-thickness endoscopic suturing approach with serial suture placement to achieve gastric volume reduction along the greater curvature while preserving the gastric fundus, consistent with contemporary ESG technical standards and bariatric endoscopy practice recommendations.

All patients undergoing ESG were enrolled in a standardized, multidisciplinary post-procedure follow-up program. This program included longitudinal care with clinical team members, consisting of an endoscopist, endocrinologist or obesity medicine specialist, and/or the patient’s primary care physician, in addition to nutritional counseling and behavioral support as indicated. Routine follow-up visits were recommended at regular intervals during the first year after ESG, with continued periodic follow-up thereafter, consistent with institutional practice for chronic obesity management.

Patients were encouraged to maintain ongoing engagement with the clinical team beyond the early post-procedural period. While a minimum follow-up schedule was recommended to all patients, individuals could elect to have more frequent clinical contact based on patient preference, clinical need, weight trajectory, or provider recommendation. Follow-up encounters focused on weight monitoring, dietary adherence, symptom assessment, and adjustment of adjunctive therapies when appropriate.

### Exercise assessment

Physical activity counseling was provided to all patients as part of routine post-ESG care. Patients were encouraged to engage in regular physical activity, including a target of at least 150 min of moderate-intensity aerobic exercise per week, in accordance with standard obesity management recommendations. Exercise participation was assessed retrospectively through clinician documentation in follow-up notes and classified based on recorded patient report of adherence to a structured or regular exercise regimen during the study period.

### Participant candidacy

Eligible participants were adults aged 18 years or older who had completed at least two years of post-procedure follow-up and had available data on weight trajectory and behavioral parameters, including exercise participation and clinic visit frequency. Patients were excluded if they had missing key anthropometric or behavioral variables, a history of prior bariatric or foregut surgery, pregnancy during the follow-up period, or medical conditions known to significantly influence body weight independent of the procedure, such as active malignancy, untreated thyroid disease, or chronic corticosteroid therapy.

### Data collection and variables

Demographic, clinical, and behavioral data were extracted from the electronic medical record. Baseline variables included age, sex, race, body mass index (BMI), weight, and obesity-related comorbidities such as hypertension, type 2 diabetes, and dyslipidemia. Behavioral variables included documentation of structured exercise participation and use of anti-obesity pharmacotherapy during follow-up. Exercise participation was assessed retrospectively through clinician documentation in follow-up notes and classified based on recorded patient report of adherence to a structured or regular exercise regimen during the study period. Structured exercise generally referred to planned, repetitive physical activity performed on a consistent basis, including aerobic exercise, resistance training, or supervised fitness programs, in accordance with routine obesity management counseling. Objective measures of exercise frequency, intensity, and duration were not consistently available.

### Assessment of follow-up intensity

Post-procedure clinical follow-up was assessed by counting the number of in-person or telemedicine visits with the multidisciplinary weight management team (i.e., clinician, dietitian, psychologist) during the observation period. These encounters represented routine visits for post-ESG care, including nutritional counseling, behavioral reinforcement, and medical evaluation. To evaluate the role of structured follow-up, participants were classified according to the number of clinician follow-up visits (defined as a visit with a physician, nurse practitioner, or physician assistant). Based on the distribution of visits within the cohort, participants were grouped as having close follow-up (i.e., ≥ 5 clinical visits) or limited follow-up (i.e., < 5 clinical visits).

To ensure that the close follow-up group reflected consistent longitudinal engagement rather than clustered early visits, patients classified as having close follow-up were required to have no gap exceeding six months between consecutive clinician visits during the follow-up period. This criterion was applied to distinguish sustained follow-up intensity from episodic or sporadic care and to better capture meaningful ongoing clinical engagement over time. The ≥ 5-visit threshold was chosen to reflect a clinically meaningful distinction between patients engaged in consistent longitudinal care and those with less frequent clinician contact.

### Outcome measures

The primary outcome was the percentage of total body weight loss (TBWL%) at the most recent available follow-up visit. TBWL was calculated to quantify the proportional reduction in weight relative to baseline using the following formula:

$$ {\text{TBWL }}\left( \% \right) = \left[ {\left( {{\text{Baseline weight}}{-}{\text{Follow - up weight}}} \right)/{\text{Baseline weight}}} \right] \times 100 $$

This measure was selected to standardize weight change across patients with varying baseline body weights and to enable comparison with previously published ESG studies.

The secondary outcome was the association between engagement in a structured exercise regimen and TBWL%.

### Statistical analysis

Continuous variables were summarized as means and standard deviations, and categorical variables as frequencies and percentages. Between-group comparisons were performed using independent-sample *t*-tests for continuous variables and chi-square or Fisher’s exact tests for categorical variables, as appropriate. Two-sided *p*-values < 0.05 were considered statistically significant. All analyses were performed using JMP Pro version 18.0 (SAS Institute Inc., Cary, NC, USA). The study followed the Strengthening the Reporting of Observational Studies in Epidemiology (STROBE) guidelines for cohort studies (Supplemental Material).

## Results

### Baseline demographic and clinical characteristics

A total of 56 patients met inclusion criteria for the analysis. The mean age of the cohort was 49.6 ± 11.3 years, and the majority were female (82.1%) and White (91.1%). The mean duration of post-procedure follow-up was 29.0 ± 6.0 months, reflecting sustained longitudinal engagement after ESG. Baseline demographic and clinical characteristics are presented in Table 1. There were no statistically significant differences between the close (≥ 5 visits) and limited (< 5 visits) physician follow-up groups with respect to age, sex, race, baseline weight, BMI, or the prevalence of obesity-related comorbidities, including hypertension, diabetes, and hyperlipidemia. Similarly, the use of anti-obesity pharmacotherapy, dietitian visit frequency, behavioral psychology visit frequency, and the proportion of patients who reported adherence to a structured exercise regimen did not differ significantly between groups. In contrast, the type of clinician providing longitudinal follow-up differed significantly between groups (*p* < 0.001), with patients in the close follow-up group more commonly followed by the endoscopist, whereas those in the limited follow-up group were more frequently managed by endocrinology or primary care.

**Table 1:** Baseline demographic, clinical, and behavioral characteristics stratified by follow-up visit frequency in a weight management cohort

Variable	Total	≥ 5 visits	< 5 visits	*p*-Value
Visits number, mean (%)	7.7 (5.5)	10.5 (5.1)	3.0 (1.1)	< 0.001
Demographic data				
Number of patients (%)	56	35	21	
Sex, female (%)	46 (82.1%)	29 (82.9)	17 (81.0)	1.0
Age, mean (SD)	49.6 (11.3)	49.5 (10.7)	48.1 (12.5)	0.67
Race, white (%)	51 (91.1%)	32 (91.4)	19 (90.5)	0.32
Baseline clinical parameters				
Weight, kg (SD)	102.5 (18.2)	101.2 (17.9)	102.5 (20.3)	0.80
Body mass index, kg/m^2^ (SD)	36.8 (5.0)	36.5 (5.4)	36.9 (4.7)	0.76
Hypertension, yes (%)	31 (55.4%)	20 (57.1)	11 (64.7)	0.77
Diabetes, yes (%)	9 (16.1%)	8 (22.9)	1 (5.9)	0.24
Hyperlipidemia, yes (%)	27 (48.2%)	17 (48.6)	10 (58.8)	0.56
Weight management parameters				
Use of anti-obesity medication, yes, (%)	2 (3.6%)	1 (2.9)	1 (5.9)	1.0
Incorporated exercise regimen, yes (%)	45 (80.4%)	29 (82.9)	16 (76.2)	0.73
Dietician visits, mean (SD)	2.3 (2.3)	2.2 (2.3)	2.7 (2.4)	0.43
Behavioral psychology visits, mean (SD)	1.7 (2.1)	1.3 (1.1)	1.9 (3.1)	0.37
Follow-up team, *n* (%)				< 0.001
Endoscopist	27 (48.2)	24 (70.6)	3 (14.3)	
Endocrinologist	24 (39.2)	7 (20.6)	15 (71.4)	
Endoscopist and endocrinologist	5 (8.9)	3 (8.8)	2 (9.5)	
Primary care physician	1 (1.8)	0 (0)	1 (4.8)	
Follow-up period, months	29.0 (6.0)	28.5 (5.9)	30.3 (6.1)	0.28

### Impact of physician follow-up on weight loss outcomes

**Fig. 1:**
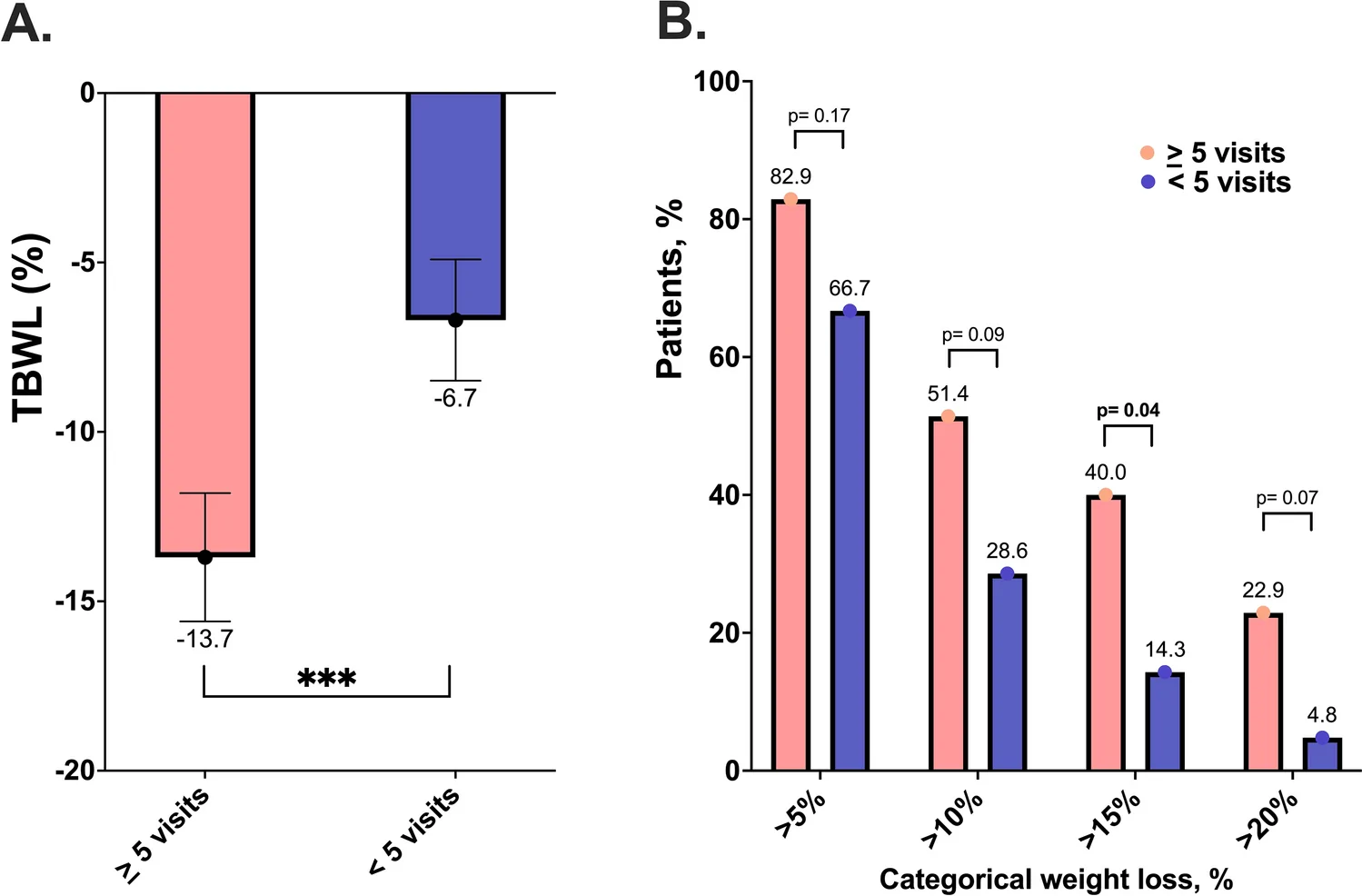
TBWL% (**A**) and categorical weight loss % (**B**) in patient with ≥ 5 vs. < 5 clinic visits. (***: *p* < 0.01)

Patients with close physician follow-up (≥ 5 visits) demonstrated greater reductions in body weight compared with those who had limited physician follow-up (< 5 visits). The TBWL% was − 13.7 ± 11.2% in the close follow-up group and − 6.7 ± 8.2% in the limited follow-up group (95% CI − 12.2 to − 1.8; *p* = 0.01) (Fig. 1A). On multivariate linear regression controlling for age, sex, race, BMI, exercise, and dietitian and behavioral psychology visits, the number of visits with clinicians was significantly associated with TBWL (*p* = 0.028).

Categorical analysis of TBWL showed a graded distribution across weight loss thresholds (Fig. 1B). Among patients with close physician follow-up, 82.9% achieved ≥ 5% TBWL compared with 66.7% in the limited follow-up group (*p* = 0.17). Similarly, 51.4% versus 28.6% achieved ≥ 10% TBWL (*p* = 0.09), 40.0% versus 14.3% achieved ≥ 15% TBWL (*p* = 0.04), and 22.9% versus 4.8% achieved ≥ 20% TBWL (*p* = 0.07). The pattern across these categories indicated progressively higher proportions of patients reaching greater degrees of weight loss in the close follow-up group compared with those with fewer physician visits.

### Impact of exercise participation on weight loss

**Fig. 2:**
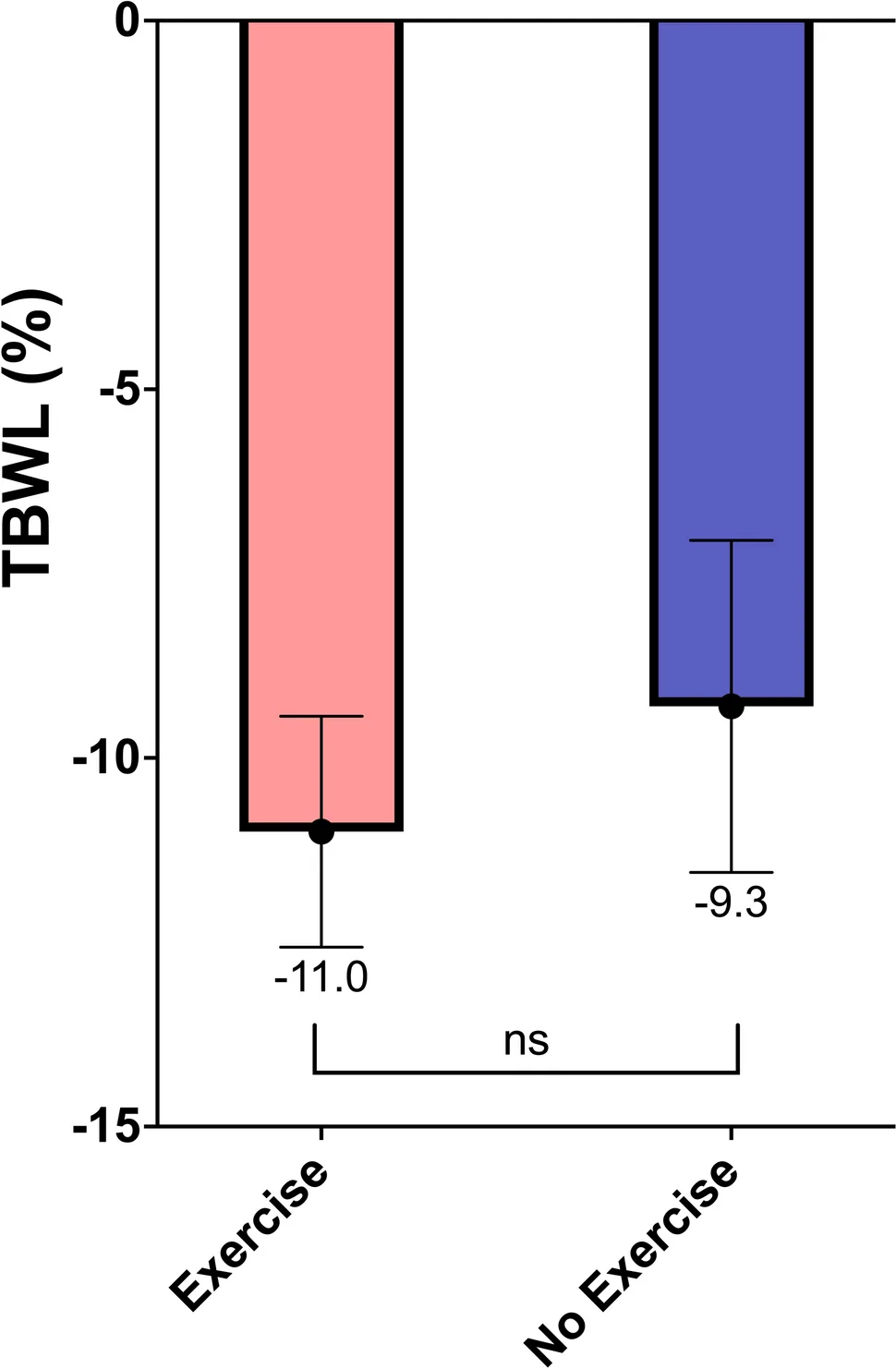
Comparison of total body weight loss (TBWL%) by exercise participation status after ESG

Exercise participation data were available for all patients included in the cohort. Overall, 80.4% of participants reported engagement in a structured exercise regimen during the follow-up period. Patients who participated in exercise achieved a mean total body weight loss (TBWL%) of − 11.0 ± 11.4%, whereas those who did not report regular exercise achieved − 9.3 ± 10.8% (95% CI − 7.2 to 3.9; *p* = 0.55) (Fig. 2). The distribution of weight loss values was comparable between groups, and no statistically significant difference in mean TBWL% was observed.

### Dietitian and behavioral psychology visits

The frequency of dietitian and behavioral psychology visits did not differ significantly between patients with close clinician follow-up and those with limited follow-up. Patients with close follow-up had a mean of 2.2 ± 2.3 dietitian visits compared with 2.7 ± 2.4 visits in the limited follow-up group (*p* = 0.43). Similarly, behavioral psychology visits were comparable between groups (1.3 ± 1.1 vs. 1.9 ± 3.1 visits, *p* = 0.37), indicating no significant between-group differences in these potential confounding supportive care inputs.

## Discussion

In this retrospective cohort of patients who underwent ESG with long-term follow-up, we found that the frequency of clinician follow-up visits was strongly associated with weight outcomes. Patients who maintained close, longitudinal engagement with their clinician team achieved roughly twice the magnitude of total body weight loss compared with those who attended fewer follow-up visits. A similar trend was observed when weight loss was categorized into clinically meaningful thresholds, with progressively higher success rates among those with more consistent clinician contact. Although exercise participation was common in this cohort, its contribution to weight reduction was modest and did not reach statistical significance. Collectively, these findings underscore the pivotal role of structured medical follow-up in sustaining the benefits of ESG and extend the understanding of behavioral and procedural determinants of long-term success.

These results highlight that ESG outcomes are not solely determined by the technical success of the procedure but also by the intensity of ongoing clinical engagement. Consistent clinician follow-up likely reinforces adherence to dietary recommendations, facilitates early identification of weight plateaus, and provides opportunities for timely behavioral or pharmacologic adjustments [25, 26]. Importantly, the high intensity arm did not have higher obesity medication use, to which we otherwise could have attributed the superior weight loss. Notably, differences in weight outcomes were not explained by more frequent dietitian or behavioral psychology visits, as visit frequency for both allied health services was similar between follow-up groups and was not independently associated with greater TBWL. In addition to our findings, prior literature has also emphasized the importance of structured follow-up in determining ESG outcomes. In an early prospective study, Lopez-Nava et al. demonstrated that, after adjusting for baseline BMI, the number of nutritional and psychological follow-up contacts independently predicted greater total body weight loss at one year [23]. Similarly, in a larger single-center analysis of 962 patients undergoing various endoscopic bariatric therapies, the same group reported that adherence to multidisciplinary follow-up, rather than procedure type, was the principal determinant of weight loss at one year [27]. These data parallel our observations, reinforcing that longitudinal engagement with the medical and behavioral care team is a key modifiable factor driving sustained weight reduction after ESG.

The observed trend toward greater weight loss among exercisers, though not statistically significant, is consistent with data demonstrating that regular physical activity enhances energy expenditure, preserves lean mass, and contributes to long-term weight stability [28, 29]. However, the lack of significance in this study may reflect heterogeneity in exercise intensity, adherence, and documentation, as well as limited power to detect small between-group differences. Larger studies with objectively measured activity metrics are warranted to clarify the independent contribution of exercise in the ESG setting.

### Clinical implications

These findings have several practical implications. First, they reinforce that the durability of ESG outcomes depends on the quality and continuity of aftercare. Structured follow-up with the physician, whether in person or via telemedicine, should be considered an integral part of ESG programs and not an optional adjunct [30]. Second, centers implementing ESG should consider embedding formal follow-up protocols that include scheduled physician encounters, nutritional counseling, and behavioral reinforcement. Third, from a systems perspective, ensuring accessibility to long-term follow-up, through hybrid or digital models, may enhance equity and sustainability of outcomes. Future research should focus on determining the optimal frequency and structure of post-ESG visits, as well as evaluating cost-effectiveness and patient adherence patterns.

### Strengths and limitations

This study has several strengths, including its real-world design, extended follow-up duration, and inclusion of both clinical and behavioral variables. The use of a well-defined follow-up classification and objective anthropometric outcomes strengthens internal validity. However, several limitations should be acknowledged. The retrospective, single-center design limits causal inference and generalizability. Moreover, the relatively low use of anti-obesity pharmacotherapy in this cohort may limit generalizability to contemporary obesity management practice, particularly given the increasing use of GLP-1 receptor agonists and dual incretin-based therapies alongside ESG. Future studies evaluating the interaction between structured follow-up, adjunctive pharmacotherapy, and long-term ESG outcomes in modern multimodal treatment paradigms are warranted. Exercise participation was self-reported and derived from retrospective clinician documentation rather than objectively measured through validated activity instruments or wearable monitoring devices. As a result, differences in exercise intensity, duration, frequency, and adherence could not be reliably quantified, introducing the potential for recall and reporting bias. Selection bias is an important consideration in interpreting our findings. By including only patients with at least two years of follow-up and complete behavioral data, our cohort may preferentially represent individuals who were more engaged with care, which itself is associated with improved weight loss outcomes. In addition, reverse causation cannot be excluded, as patients experiencing greater weight loss may have been more motivated to maintain follow-up. We were unable to reliably capture the total number of ESG procedures performed during the study period or fully characterize excluded patients, limiting assessment of selection effects. Early post-procedural complications were not systematically associated with increased long-term follow-up in this cohort; however, residual confounding related to clinical need for follow-up cannot be excluded. Accordingly, our findings should be interpreted as demonstrating an association rather than a causal relationship between clinician follow-up intensity and weight loss outcomes. Additionally, unmeasured confounders such as patient motivation, socioeconomic status, healthcare access, geographic distance from the treatment center, and other barriers to longitudinal engagement may have influenced both follow-up adherence and weight loss outcomes. Both in-person and telemedicine encounters were included in the assessment of clinician follow-up intensity; however, the relative impact of visit modality on long-term outcomes could not be evaluated in this study. Finally, adverse events were not systematically evaluated as part of the current analysis, as the primary objective of the study was to examine the relationship between longitudinal follow-up engagement and long-term weight loss outcomes. In addition, the relatively small sample size limited the ability to perform meaningful correlation analyses between procedural adverse events, follow-up intensity, and weight outcomes. Future studies incorporating standardized safety outcome assessment are warranted.

## Conclusion

In this real-world cohort of patients undergoing endoscopic sleeve gastroplasty with extended follow-up, higher frequency of physician follow-up visits was independently associated with greater long-term weight loss. Exercise participation showed a positive, though nonsignificant, trend toward improved outcomes. Together, these findings underscore that procedural efficacy alone does not determine success after ESG; sustained, structured follow-up within a multidisciplinary framework is essential for achieving and maintaining meaningful weight reduction. Establishing standardized post-ESG care pathways that prioritize regular physician engagement and behavioral reinforcement may enhance durability of outcomes and should be considered a core component of future obesity management models.

## Supplementary Information

Below is the link to the electronic supplementary material.

Supplementary file1 (DOCX 31 kb)
